# A Novel Sex-Dependent Target for the Treatment of Postoperative Pain: The NLRP3 Inflammasome

**DOI:** 10.3389/fneur.2019.00622

**Published:** 2019-06-12

**Authors:** Ashley M. Cowie, Bonnie N. Dittel, Cheryl L. Stucky

**Affiliations:** ^1^Department of Cell Biology, Neurobiology, and Anatomy, Medical College of Wisconsin, Milwaukee, WI, United States; ^2^Blood Research Institute, Versiti, Milwaukee, WI, United States; ^3^Department of Microbiology and Immunology, Medical College of Wisconsin, Milwaukee, WI, United States

**Keywords:** NLRP3, interleukin-1β, sex differences, pain, tissue injury, innate immunity

## Abstract

In recent years the innate immune system has been shown to be crucial for the pathogenesis of postoperative pain. The mediators released by innate immune cells drive the sensitization of sensory neurons following injury by directly acting on peripheral nerve terminals at the injury site. The predominate sensitization signaling pathway involves the proinflammatory cytokine interleukin-1β (IL-1β). IL-1β is known to cause pain by directly acting on sensory neurons. Evidence demonstrates that blockade of IL-1β signaling decreases postoperative pain, however complete blockade of IL-1β signaling increases the risk of infection and decreases effective wound healing. IL-1β requires activation by an inflammasome; inflammasomes are cytosolic receptors of the innate immune system. NOD-like receptor protein 3 (NLRP3) is the predominant inflammasome activated by endogenous molecules that are released by tissue injury such as that which occurs during neuropathic and inflammatory pain disorders. Given that selective inhibition of NLRP3 alleviates postoperative mechanical pain, its selective targeting may be a novel and effective strategy for the treatment of pain that would avoid complications of global IL-1β inhibition. Moreover, NLRP3 is activated in pain in a sex-dependent and cell type-dependent manner. Sex differences in the innate immune system have been shown to drive pain and sensitization through different mechanisms in inflammatory and neuropathic pain disorders, indicating that it is imperative that both sexes are studied when researchers investigate and identify new targets for pain therapeutics. This review will highlight the roles of the innate immune response, the NLRP3 inflammasome, and sex differences in neuropathic and inflammatory pain.

## Introduction

A unique combination of molecular and cellular factors can lead to acute and chronic pain conditions with varying pathologies. Despite this, pain is categorized into the following broad categories: inflammatory, neuropathic, and syndrome-based (e.g., fibromyalgia). There is overlap between these generalized categories. For example, inflammation can result in nerve damage, nerve injury involves inflammation, and syndrome-based pain can be neuropathic or inflammatory or both. Inflammatory pain occurs with peripheral tissue damage and the resulting tissue inflammation. Alternatively, neuropathic pain results from direct damage to nerves in the peripheral or central nervous systems. Postoperative pain has both inflammatory and neuropathic qualities (1). It is widely recognized that postoperative pain occurs as a result of the direct cutting of tissues and peripheral nerves at the surgical site.

Rodent models of postoperative pain have been consistently used to study the underlying causes of postoperative pain. Rodent models of surgical pain are strong preclinical models because the injury induced in the animal and human is similar, and therefore, these models likely recapitulate patient phenotypes and mechanisms (1–3). The most common postoperative pain model involves cutting through the skin and underlying muscle (flexor digitorum brevis), which reliably produces mechanical and heat hyperalgesia at the incision site (4–9). There is a robust immune response in this model that includes infiltration of neutrophils, macrophages, and lymphocytes. The immune response aids in wound healing, but also results in sensitization of sensory neurons to mechanical and heat stimuli (1, 10–13). The immune response begins at the incision site or site of tissue damage and moves proximally to the dorsal root ganglia and spinal cord.

There is a rapidly growing body of evidence demonstrating that the development and maintenance of postoperative pain are not solely dependent on the increased excitability of sensory neurons alone at the incision site, but they also depend on immune cell interactions with sensory neurons and activation of canonical immune receptors expressed by sensory neurons. Components of the innate immune system have emerged as crucial mediators in the development and maintenance of hypersensitivity following incision. Pattern-recognition receptors (PRRs) are part of the innate immune system and are among the first to be activated in response to tissue damage; their activation is important for the induction of immune responses leading to pathogen elimination and subsequent tissue repair (14). PRRs include cytosolic NOD-like receptors (NLRs) which, when activated, form inflammasomes. The NLR protein 3 (NLRP3) inflammasome is the best characterized NLR and has been shown to be critical in driving the immune response to sterile tissue damage (15), the type of inflammation that occurs with surgical incision. Additionally, NLRP3 is known to play a role in several painful conditions that arise from sterile tissue damage (16–30). Since the immune system is known to be sexually dimorphic, much recent attention has been given to understanding the sex differences and their causative factors that underlie painful conditions. However, little is known about the effects of sex on NLRP3 or the role of NLRP3 in postoperative pain. Therefore, this review provides a new insight into the relationship between NLRP3 and postoperative pain. Here we discuss the current understanding of sexual dimorphism in the innate immune system response to tissue injury and the role it plays in inflammatory and neuropathic pain conditions by focusing on the NLRP3 inflammasome.

## The Immune Response to Incisional Injury

### Immune Cell Involvement

Surgical incision results in local tissue injury, which destroys physical barriers between the body and environment, and increases the risk of exposure to environmental and commensal microbes. These consequences of surgery all lead to activation of the innate immune system and local inflammation. Inflammation occurs immediately following tissue injury as an attempt to clear debris and initiate healing. Initially immune cells such as mast cells, neutrophils, and monocytes/macrophages are recruited to the injury site by mediators that are released in tissues, by neurons and by tissue-resident immune cells (12, 31–34). Recruitment and activation of different immune cells following injury occurs in the same sequence in both sexes. First, dermal mast cells regulate inflammation immediately following cutaneous wounding by releasing inflammatory mediators, thereby increasing vascular permeability and recruiting neutrophils (35, 36). The neutrophil recruitment is generally followed by monocyte/macrophage recruitment, which occurs 1–2 days following injury (12, 34, 37, 38). Macrophages play a dual role in wound healing, where initially they promote inflammation and then later, they switch to a reverse role where they promote the resolution of inflammation (34). Lastly, during the resolution of inflammation phase, T cells infiltrate the wound to aid in healing (34, 39).

### The NLRP3 Inflammasome and Interleukin-1β Production

Surgical trauma is aseptic and causes the release of damage-associated molecular patterns (DAMPs) (40). DAMPs are endogenous molecules that are released from damaged or dying cells and serve as a signal for tissue damage (41). Soluble DAMPs that are released as a result of incision include: heparan sulfate (42, 43), fibronectin (44, 45), hyaluronan (46–48), β-defensins (49–51), heat shock protein 70 (Hsp70) (52), and high mobility group box-1 (HMGB1) (53, 54). These DAMPs then bind to PRRs such as Toll-like Receptors (TLRs) on innate immune cells (mast cells, neutrophils, monocytes/macrophages) and sensory neurons, specifically Toll-like Receptor 4 (TLR4) (18, 49, 55–58). Stimulation of TLR4 leads to activation of the transcription factor NF-κB and upregulation of the synthesis of pro-inflammatory cytokines like interleukin-1β (IL-1β) (59). Stimulation of TLR4 also serves as the priming signal for NLRP3, the activator of IL-1β (59).

**Figure 1:**
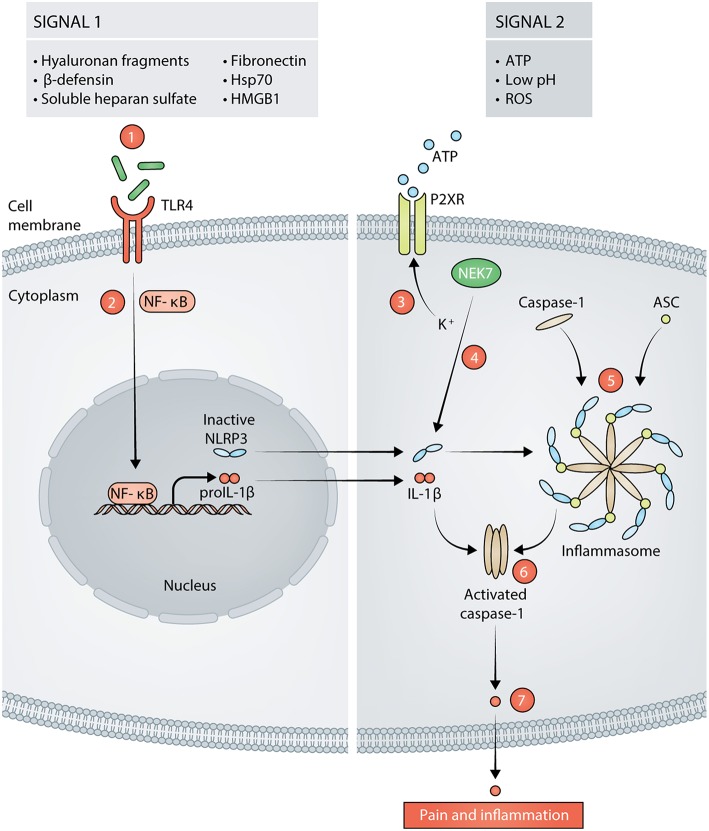
Signals in response to tissue damage activate the NLRP3 inflammasome (signal 1 and signal 2). Hyaluronan fragments, β-defensins, soluble heparan sulfate, fibronectin, 70 kilodalton heat shock proteins (Hsp70), and high mobility group box 1 (HMGB1) are released following incision and act as signal 1 for NLRP3 by stimulating TLR4 on the cell membrane (1). Stimulation of TLR4 leads to activation of NF-κB and transcription of proIL-1β and NLRP3 (2). Adenosine Triphosphate (ATP), reactive oxygen species (ROS), and low pH can then act as signal 2 for NLRP3. ATP acts on purinergic ion channel receptors (P2XR) such as P2X7 or P2X4 which results in potassium (K^+^) efflux from the cell (3). The decrease in K^+^ concentration is sensed by NIMA Related Kinase 7 (NEK7). NEK7 associates with inactive NLRP3, thereby activating it (4). Active NLRP3 then forms a scaffold with caspase-1 and apoptosis-associated speck-like protein containing a CARD (ASC), thus, forming the inflammasome (5). Caspase-1 is activated by the formation of the inflammasome (6). Activated caspase-1 cleaves proIL- into mature IL-1β that is released from the cell and subsequently, results in pain and inflammation (7).

NLRP3 is predominately expressed by cells in lymphoid organs and tissues that are highly populated by immune cells. These cells include but are not limited to mast cells, neutrophils, macrophages, monocytes, dendritic cells, and neurons in both the peripheral and central nervous systems (29, 60–62). The expression of NLRP3 in these cell types must be induced by inflammatory stimuli, which prevents uncontrolled release of IL-1β. NLRP3 requires two signals for canonical activation and for IL-1β secretion: the first signal primes the cell to express NLRP3 and pro-IL-1β, and the second signal induces inflammasome assembly and activation (41, 63, 64). NLRP3 forms a scaffold with apoptosis-associated speck-like protein containing a CARD (ASC) to provide a molecular platform for activation of pro-caspase-1, which collectively comprises the inflammasome (65). Activated caspase-1 cleaves pro-IL-1β into active IL-1β, which is secreted. Several DAMPs that are present after incision and that can serve as the activation signal for NLRP3 inflammasome assembly include: ATP (66, 67), reactive oxygen species (ROS) (68, 69), and low pH (70–72). The activation cascade for NLRP3 is summarized in Figure 1. Indeed, the presence of priming and activating DAMPs for NLRP3 activation after aseptic tissue injury implicate a role for NLRP3 in mediating the postoperative pain phenotype. We recently showed that NLRP3 is upregulated at the surgical site and drives postoperative mechanical pain-like behaviors in male mice, but not in female mice (13). This study provided the first evidence that NLRP3 drives postoperative pain and revealed that the immune-mediated mechanisms that underlie postoperative pain are sex-specific.

## Sexual Dimorphism in the Immune Response

The importance of taking sex into consideration when studying painful injuries and their underlying mechanisms was recently highlighted when it was revealed by Sorge et al. that male mice require microglia and TLR4, whereas female mice require T cells to mediate chronic neuropathic pain (73, 74). In addition, hormones significantly contribute to sex-based differences in the immune response (75). Estradiol, progesterone, and testosterone are the primary hormones that affect the immune response. Female vertebrates have higher baseline estrogen and progesterone levels whereas male vertebrates have higher baseline testosterone levels. Estrogen, progesterone, and testosterone receptors are expressed on both adaptive (T cells and B cells) and innate (macrophages, dendritic cells, neutrophils, and natural killer cells) immune cells; the effects of hormones on these receptors are dose-dependent (76). Consequently, there are alterations in immune system function during pregnancy, menses, and menopause. Each of the three hormones mentioned above affects the immune system during injury or disease states in different ways, and therefore, the immune response to injury differs between males and females.

The level of immune cell infiltration and the extent of the innate immune response at an injury site are both affected by sex hormones. Estrogen suppresses mast cell release of histamine and as a result, fewer neutrophils are recruited to a wound site in females (77, 78). In regards to the effects of estrogen on the macrophage response to injury, Price et al. recently showed that a reduced number of macrophages is recruited to a postoperative tissue site in female mice compared to male mice (79). Additionally, high estrogen levels skew macrophages toward the M2 phenotype (anti-inflammatory) while high testosterone levels promote the M1 phenotype (proinflammatory). As a consequence of the M1 phenotype, males have higher expression of TLR4, NLRP3, and produce more IL-1β than females (75, 80–83). However, chronic estrogen exposure induces increased TLR4-mediated production of IL-1β in macrophages (84). Despite the lower levels of immune cell infiltrate within a wound in females as compared to males, cytokine levels in females are sustained longer than in males, and females have more tissue-resident immune cells than males (75, 78). Furthermore, data from our laboratory demonstrated that males have more IL-1β protein at the peri-incisional site than females (13). Whereas, we showed that NLRP3 mRNA was upregulated by incision to a similar extent in males and females, global deletion of NLRP3 decreased IL-1β levels and sensitization to mechanical stimuli only in males. This suggested that NLRP3 may be differentially regulated post-transcriptionally in males and females following tissue incision, where in females, the IL-1β production occurs independent of NLRP3. The activation pathway for NLRP3 has been suggested to differ in macrophages from male and female Systemic Lupus Erythematosus patients as well (85). In addition, males and females utilize TLR4 in a cell-specific manner. Stimulation of TLR4 on macrophages drives pain in male mice whereas stimulation of TLR4 on sensory neurons drives pain in female mice (86, 87). Furthermore, fibroblasts which play critical roles in the immune response and local environment during tissue injury, also produce IL-1β, and fibroblast IL-1β levels are differentially affected by testosterone and estrogen treatment (88). Considering all of the evidence above for sex-driven differences in the immune response to injury and the resulting differences in sensory neurons, it is imperative to take the sex of an individual into account when selecting and assessing the efficacy of pain interventions.

## Proinflammatory IL-1β and Postoperative Pain

The primary function of IL-1β is to elicit a pro-inflammatory response to DAMPs (41). IL-1β is expressed by macrophages, monocytes, neutrophils, mast cells, glial cells, and sensory neurons (89–91). Secreted IL-1β exerts its proinflammatory effects through various mechanisms. These include increasing production of other inflammatory mediators via rapidly inducing their mRNA expression, increasing vascular permeability, recruiting immune cells, directly eliciting pain via binding of the IL-1β receptor on sensory neurons, and inducing neurogenic inflammation through sensory neuron sensitization and increased production of calcitonin gene-related peptide alpha (CGRPα) (91–94). IL-1β acts through its receptor type I IL-1 receptor (IL-1R1), which is ubiquitously expressed on neurons of the peripheral and central nervous systems (95, 96). When IL-1R1 binds IL-1β, the accessory protein IL-1R3 is recruited to induce intracellular signaling cascades via association of their intracellular Toll- and IL-1R-like (TIR) domains with signaling proteins (97). The cascade begins with the association of myeloid differentiation primary response gene 88 (MYD88) and interleukin-1 receptor–activated protein kinase (IRAK) 4 with the TIR domains. This leads to complex formation of IRAK1, IRAK2, and tumor necrosis factor–associated factor (TRAF) six and subsequent activation of transcription factors such as NF-κB to upregulate inflammatory genes.

Postoperative pain is characterized by persistent acute pain at the incisional site which is associated with release of proinflammatory cytokines, including IL-1β. Studies have found that IL-1β is significantly upregulated at the incision site (12, 13, 32, 98–101). Wolf et al. demonstrated that either systemic inhibition of IL-1β signaling by its receptor antagonist IL-1ra or deletion of IL-1R1 prevented the development and maintenance of postoperative mechanical hypersensitivity at the incision site (102). Other groups further demonstrated that inhibition of IL-1β signaling through antagonism of its receptor significantly decreased postoperative pain-like behavior in rodents (32, 98, 100). Furthermore, additional research has established that inhibition of the upstream mediators of IL-1β, such as TLR4 (98, 103), NF-κB (103), caspase-1 (104), or NLRP3 (13), decreases postoperative pain-like behaviors in rodents. General blockade of IL-1β signaling, like that obtained with FDA approved Anakinra (IL-1R1 antagonist), increases the rate of infections due to the necessity of IL-1β for bacterial infection clearance (105, 106). Whereas, inhibition of TLR4, NF-κB, and caspase-1 is more ubiquitous, inhibition of one inflammasome is more specific. Therefore, reduction of IL-1β but not complete depletion, through inhibition of only NLRP3 may avoid these complications while decreasing postoperative pain. However, inhibition of NLRP3 alone may only be effective in males but not females (13). Not only are sex differences prevalent in mice that received surgery, but they are present in human postoperative pain as well. For instance, a predicative factor of chronic postoperative pain is female sex (107–109). Thus, a therapeutic for the treatment of postoperative pain in females must target the unique factors that are required for the development of postoperative pain in females.

## The Role of NLRP3 in Pain Disorders

Much is yet to be learned about NLRP3 and postoperative pain, however a role for NLRP3 in pain disorders is emerging (13). NLRP3 has been shown to be involved in the pathogenesis of both inflammatory and neuropathic pain conditions. Inflammatory pain depends on the sensitization of nociceptive neurons by proinflammatory mediators such as IL-1β (110, 111). In an acute model of dural inflammation, the injection of an “inflammatory soup” (comprised of histamine, serotonin, bradykinin, and prostaglandin E2 at pH 5.5) resulted in activated NLRP3 and caspase-1, and increased IL-1β expression in C fiber type neurons of the trigeminal ganglia (29). The inflammatory soup injection also resulted in pain-like behaviors which were alleviated by a caspase-1 inhibitor. In another inflammatory pain model, the complete Freund's adjuvant (CFA) model, NLRP3 was shown to be activated in the skin of rats (28). Electroacupuncture following CFA injection attenuated the expression of NLRP3 and ultimately eliminated the pain-like behavior (28). Additionally, NLRP3 has been demonstrated to be crucial for the pathogenesis of rheumatoid arthritis in both humans and rodents (27, 112, 113). Further, upregulation of NLRP3 has been shown to occur in rodent models of gout, and its inhibition or deletion ameliorated the pathology and pain (24, 25, 114–116). Collectively, these data point to a key role for NLRP3 in inflammatory pain.

Neuropathic pain involves direct damage to nerves from injury or disease. IL-1β significantly contributes to traumatic neuropathic pain where its expression is upregulated in the dorsal root ganglia and spinal cord, as well as in damaged nerves in rodent models of neuropathic pain and in patients with neuropathic pain (18, 117–119). NLRP3 plays a role in various rodent models of neuropathic pain. Alleviation of sciatic nerve ligation neuropathic pain with miR-23a overexpression, or CXCR4 knockdown results in decreased NLRP3 expression (16). A study utilizing the chronic constriction sciatic nerve injury model of neuropathic pain demonstrated that NLRP3 is upregulated by nerve injury and that treatment with Peptide5, a Connexin 43 mimetic peptide that blocks hemichannels, decreased NLRP3 expression and mechanical pain-like behavior (17). In addition, chemotherapy-induced neuropathy models of neuropathic pain revealed that NLRP3 is upregulated in both oxaliplatin-induced nerve injury (19) and paclitaxel-induced nerve injury (20) models, and inhibition of NLRP3 decreased the mechanical pain-like behaviors in both models. In contrast to these findings, it was demonstrated that global knockout of NLRP3 had no effect on neuropathic pain in the spared nerve injury model of neuropathic pain (120). This is consistent with discrepant findings that challenge the view that microglia drive neuropathic pain exclusively in males (121–123). When compared, these studies demonstrate that different models of neuropathic pain (spared nerve injury, spinal nerve transection, spinal nerve ligation, and partial nerve ligation) do not produce the same findings. Together, these studies suggest that while NLRP3 contributes to a variety of etiologies of neuropathic pain it is dependent on the type of injury and the diverse factors that are likely involved in different injuries.

Although much remains to be discovered about the mechanistic causes of body-wide pain syndromes such as fibromyalgia, several studies have indicated a role for NLRP3 in fibromyalgia-associated pain, and NLRP3 was found to be upregulated in patients with fibromyalgia (21–23). Further research is needed in animal models of fibromyalgia and tissues from patients with fibromyalgia.

## Conclusion

The discovery of inflammasomes has provided new insights into the molecular mechanisms underlying the innate immune system activation in inflammatory and neuropathic pain conditions. As discussed here, many inflammatory and neuropathic pain conditions, and specifically postoperative pain, involve the innate immune system and NLRP3. Therefore, modulators of NLRP3 may provide a novel, selective, and effective pain therapeutic target. Notwithstanding, our understanding of the functional roles and the mechanisms of activation of the NLRP3 inflammasome in pain conditions is in its infancy. Additionally, it is imperative that further research be conducted on the effect of the sex of an individual on NLRP3 function since all of the rodent studies on NLRP3 in the pain conditions discussed here, except for the report by our group (13), were performed in males only. In our study we revealed that there are significant sex differences when NLRP3 is deleted, suggesting that NLRP3 plays different roles in males and females following tissue injury (13). Additionally, the literature concerning the specific immune responses to perioperative incision in males and females is insufficient and far more studies that include females must be done. Therefore, we conclude that targeting NLRP3 may provide a novel approach to control pain, but that further research needs to uncover the mechanistic differences and roles of NLRP3 in wound healing following surgery in females and males.

## Data Availability

No datasets were generated or analyzed for this study.

## Author Contributions

AMC wrote and edited the manuscript. CLS and BND edited the manuscript.

### Conflict of Interest Statement

The authors declare that the research was conducted in the absence of any commercial or financial relationships that could be construed as a potential conflict of interest.
